# A cluster of *Candida parapsilosis* displaying fluconazole-trailing in a neonatal intensive care unit successfully contained by multiple infection-control interventions

**DOI:** 10.1017/ash.2024.77

**Published:** 2024-05-16

**Authors:** Hiroaki Baba, Hajime Kanamori, Asami Nakayama, Takami Sato, Makoto Katsumi, Takae Chida, Shinobu Ikeda, Rio Seki, Teppei Arai, Katsuhiko Kamei, Koichi Tokuda

**Affiliations:** 1 Department of Infectious Diseases, Internal Medicine, Tohoku University Graduate School of Medicine, Sendai, Miyagi, Japan; 2 Division of Infection Control, Tohoku University Hospital, Sendai, Miyagi, Japan; 3 Department of Laboratory Medicine, Tohoku University Hospital, Sendai, Miyagi, Japan; 4 Medical Mycology Research Center, Chiba University, Chiba, Japan

## Abstract

**Objective:**

This study aimed to investigate and contain a cluster of invasive candidiasis cases caused by fluconazole-resistant *Candida parapsilosis* (FRC) in a neonatal intensive care unit.

**Methods:**

Active surveillance was initiated. Direct observations of hand-hygiene compliance (HHC) among staff were conducted before and after the implementation of hand-hygiene (HH) education. Thirty-five environmental cultures were obtained. Phylogenetic analysis of FRC was performed using Fourier-transform infrared spectroscopy and microsatellite genotyping.

**Results:**

A total of 14 patients (mean birth weight = 860 g, gestational age = 25 weeks) infected with FRC were identified using the fully automated analyzer, including 5 with clinical infection (three with catheter-related bloodstream infection, one with cutaneous infection, and one with fatal peritonitis) and 9 with colonization. The HHC rate in nurses before performing a sterile or aseptic procedure significantly improved after the HH education (*P* < .05). Sinks near the patients were contaminated with FRC. All FRC strains were confirmed to be susceptible to fluconazole using the CLSI method, and the microdilution procedure indicated a trailing effect. Phylogenetic analysis showed that all the fluconazole-trailing isolates from patients were clustered together and had the same genotype. Sinks were successfully decontaminated using accelerated hydrogen peroxide and drainage pipes were replaced. Ultraviolet-C decontamination was applied in the milk preparation room. No new cases were detected after the education and disinfection interventions.

**Conclusions:**

Sinks are an important reservoir of *C. parapsilosis*. Active surveillance, environmental hygiene, and constant staff education on maintaining a high level of HHC are necessary to limit the spread of *C. parapsilosis*.

## Introduction

Invasive candidiasis (IC) is a leading cause of morbidity and mortality in neonatal intensive care units (NICU).^1^ The primary risk factor for IC is prematurity, complemented by the disruption of epithelial barriers caused by procedures and equipment, such as endotracheal intubation and central venous catheters.^1^ Advancements in these therapeutic procedures have significantly improved survival in premature infants; however, there has been a noticeable increase in the incidence of IC in NICU.^2^

*Candida parapsilosis* is the most common *Candida* species responsible for IC in pediatric patients.^3^ Fluconazole is one of the main therapeutic options for infections caused by *C. parapsilosis* since this pathogen exhibits an intrinsically reduced susceptibility to echinocandins.^4^ Thus, the emergence of fluconazole-resistant strains poses a significant clinical concern worldwide.^4^ Between April 2019 and March 2020, we encountered a cluster of IC cases caused by fluconazole-resistant *C. parapsilosis* (FRC) in the NICU of a tertiary hospital in Japan. This study aimed to investigate and contain a cluster of IC cases caused by FRC in the NICU.

## Methods

The clinical setting, population characteristics, and infection-control interventions associated with this cluster are summarized in the Supplementary Table 1.^5^

### Active surveillance

The tertiary hospital had an NICU with 15 beds comprising 9 in NICU 1 and 6 in NICU 2, a growing care unit (GCU) with 17 beds, and a milk preparation room. Patients newly admitted to the NICU are initially placed in NICU 1 and then transferred to NICU 2 upon stabilization from the acute phase. A cluster was first suspected in April 2019 with two cases of IC from a catheter-related bloodstream infection (BSI) caused by FRC (minimum inhibitory concentration [MIC], 32 μg/mL). This detection was made using a fully automated analyzer (RAISUS® S4, Nissui, Tokyo, Japan). The incident of unusual cases of FRC infection, which had never previously occurred in the hospital, led to the initiation of active surveillance screening for FRC among all NICU patients. This screening included admission and weekly cultures from rectal, nasal, and skin samples because these sites are commonly colonized by *Candida* species.^1,6^ The surveillance involved an epidemiologic investigation including a detailed review of medical records of patients infected or colonized with FRC. Additionally, a comparative analysis of the incidence rates of IC caused by *Candida* species other than FRC, defined as the number of IC cases per total inpatients during the same period, was conducted for three distinct periods: before the cluster (April 2015 to March 2019), during the cluster (April 2019 to March 2020), and after the cluster (April 2020 to March 2021).

### Hand hygiene education

Education for NICU staff to improve hand-hygiene compliance (HHC) was implemented in July 2019. These included informing all the NICU staff of the IC cluster caused by FRC; conducting formal lectures on the proper utilization of hand-cleaning agents, proper handwashing techniques, and the importance of hand hygiene (HH), implementing a brainstorming session to enhance adherence to appropriate HH practices. In addition, training was conducted to ensure the implementation of standard precautions^7^; however, patient/staff cohorting was not feasible due to spatial constraints and a lack of adequate staffing in the wards. Throughout the periods before, during, and after the cluster, the alcohol-based hand rub was recommended, and the NICU staff was consistently equipped with and utilized alcohol hand sanitizers. Concurrently, the wearing of artificial nails was strictly prohibited.

At the tertiary hospital, direct observations of HHC among the NICU staff, following the World Health Organization (WHO)’s five moments for HH,^8^ had been routinely conducted twice a year by four trained infection-control nurses. The assigned nurses document every HH opportunity, identifying how many medical personnel (eg, physicians or nurses) performed HH and whether the procedure was performed. The HHC rate was calculated by dividing the number of observed HH actions by the total number of opportunities. During the study period, these observations were made before (July 28, 2019) and after (November 27, 2019) implementation of the education, and after the cluster (May 19, 2020, and November 19, 2020). Feedback on HHC was provided to the NICU staff on July 5, 2019. HHC rates observed at each time point were compared using the data recorded on May 18, 2018, as a baseline.

### Environmental investigation

Despite the implementation of HH education, cases of FRC carriage and infection continued to be detected. A case-control study conducted on all patients admitted to the NICU from April to August 2019 revealed that the consumption of donor milk was associated with an increased risk of infection or colonization with FRC (odds ratio 7.20; 95% confidence interval, 1.28–40.37) (Supplementary Table 2). Based on the results, the infection-control team identified the milk preparation room, used for donor milk preparation, as a potential high-risk site for cross-contamination and subsequently conducted an environmental investigation. Overall, 35 environmental cultures were obtained from 35 sites using eSwab collection kits (Copan, Brescia, Italy). Ten of these cultures were from 5 high-touch surfaces (medical cart, infusion preparation table, refrigerated drug storage, garbage container, and computer keyboard) and 5 medical devices (ultrasound probe, weighing scale, diaper scale, medical tape, and radiation protection apron) in the NICU; 11 were from 2 high-touch surfaces (vital signs monitor and infusion pump control panel), 5 medical devices (stethoscope, axillary thermometer, bag valve mask, and ventilator monitor/tube and water trap) and an infant incubator (access port door handle, internal wall, humidifier reservoir, and external tray) in the immediate environment of a patient with spontaneous intestinal perforation; 2 were from 2 high-touch surfaces (formula preparation counter and breastmilk refrigerator) in the milk preparation room; and 12 were from 12 sink surfaces and drains (6 in the NICU, 3 in the GCU, 1 in the nurse station, and 2 in the milk preparation room). Samples were collected according to the manufacturer’s instructions.

Before the cluster, nurses in the NICU routinely cleaned high-touch environmental surfaces with disinfectant wipes containing quaternary ammonium compounds every 8 hours, although the cleaning was not performed thoroughly. The standard sinks with splash guards were used exclusively for washing hands and were cleaned daily using a neutral detergent (Supplementary Figure). Infusions, including total parenteral nutrition, are prepared exclusively on the infusion preparation table, and discarded in a sink used only for infusion disposal at the nurse station after use or upon expiration.

### Identification of fungal strains and antifungal susceptibility evaluation

Fungal strains were detected from the clinical and environmental samples using CHROMagar™ Candida media (Kanto Chemical Co., Tokyo, Japan) (Supplementary Method 1). Species identification was performed using a VITEK-MS (Sysmex-bioMérieux Japan, Tokyo, Japan). The antifungal susceptibility of *C. parapsilosis* isolates was determined using an automated analyzer. After the cluster, this susceptibility was further confirmed using the Clinical and Laboratory Standards Institute (CLSI) broth microdilution method, as described in CLSI document M27-E4.^9^

### Phylogenetic analysis

Phylogenetic analysis of FRC isolated from patients and environmental samples, along with one standard strain of *C. parapsilosis* (ATCC 22019) and two epidemiologically unrelated clinical isolates from the tertiary hospital, was performed using the IR Biotyper system with IR Biotyper client software Ver.3.1 (Bruker Daltonics GmbH & Co. KG), which is a typing machine based on Fourier-transform infrared (FT-IR) spectroscopy. The samples were prepared according to the manufacturer’s instructions.^10^ Same strains were measured in two independent biological replicates. A dendrogram was generated using single spectrum with the Exploration method: Euclidean_average_linkage. The clustering cutoff value was calculated automatically using the software, which was the result of Simpson’s index of diversity. Microsatellite genotyping was performed on all FRC isolates from patient and environmental samples as described previously (Supplementary Method 2).^11^

### Statistical analysis

Fisher’s exact test was used to compare the categorical variables. Analysis was performed using JMP Pro 16 statistical analysis software (SAS Institute, 2021). A *P*-value <.05 was considered statistically significant.

## Results

### Active surveillance

**Figure 1.**
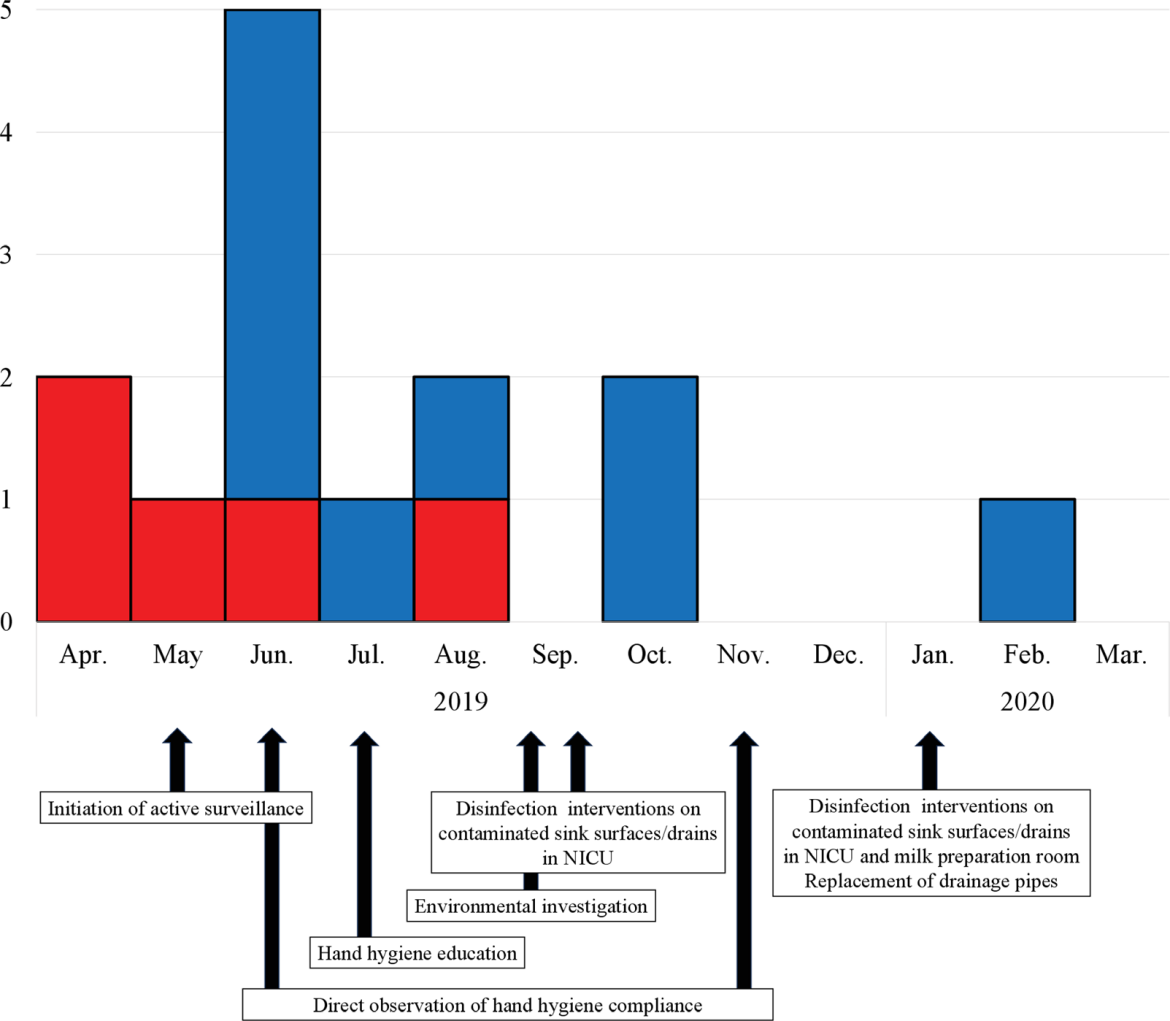
Epidemic curve of patients newly infected/colonized with fluconazole-resistant *Candida parapsilosis* (FRC) in the neonatal intensive care unit from April 2019 to March 2020. The red and blue bars indicate patients infected and colonized with FRC, respectively.

**Figure 2.**
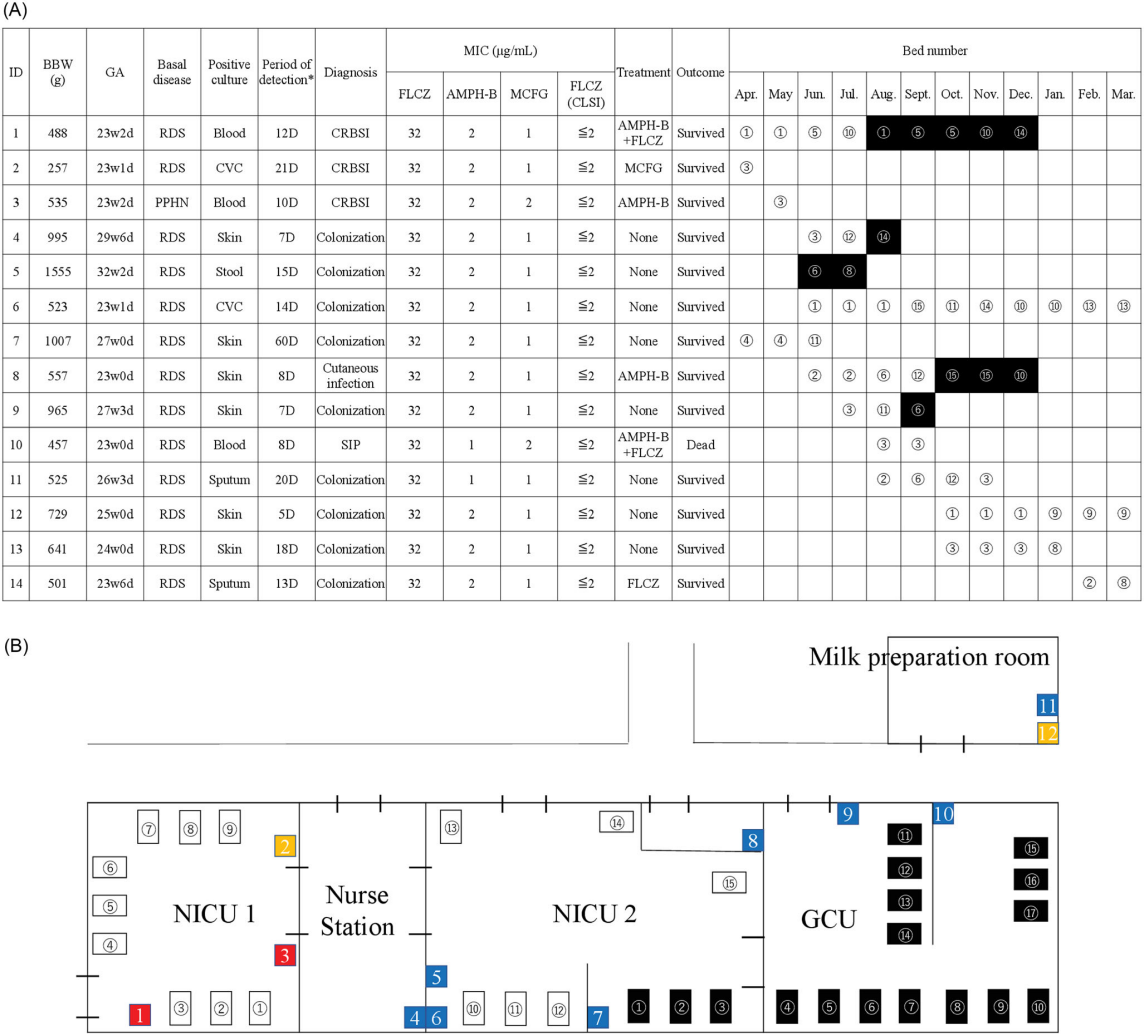
(A) Patient characteristics and locations and (B) schematic map of the neonatal intensive care unit (NICU). The circled numbers in white and black in A and B correspond to one another. In B, white and black rectangles represent the beds in the NICU and the growing care unit (GCU), respectively. Red, yellow, and blue squares in B represent fluconazole-resistant *Candida parapsilosis*-positive, fluconazole-susceptible *C. parapsilosis*-positive, and negative sinks, respectively. *Note*: *The period of detection, quantified in days (D), between the patient’s admission to the NICU and the initial detection of fluconazole-resistant *C. parapsilosis* in either clinical specimens or through active surveillance. Abbreviations: NICU, Neonatal intensive care unit; GCU, growing care unit; CVC, central venus catheter; CRBSI, Catheter-related bloodstream infection; SIP, spontaneous intestinal perforation; AMPH-B, Amphotericin B; FLCZ, fluconazole; MCFG, micafungin; CLSI, the Clinical and Laboratory Standards Institute

Among the 201 newborns (mean birth weight = 2,134 g; gestational age = 34 weeks) admitted to the NICU between May 2019 and March 2020, a total of 14 patients were identified with FRC, including 5 with clinical infection and 9 with colonization detected through active surveillance (Figure 1). Among the 14 patients infected with FRC (mean birth weight = 860 g, gestational age = 25 weeks), 5 extremely low birthweight infants were infected with FRC strains, including 3 cases of catheter-related BSI, 1 of cutaneous infection, and 1 of fatal peritonitis with intestinal perforation despite surgical and antifungal treatment with a fluconazole plus amphotericin B combination (Figure 2). After November 2019 following the introduction of HH education and disinfection interventions on sink surfaces and drains and in the milk preparation room, only one additional case occurred in February 2020. Fluconazole-resistant *Candida* isolates have not been identified from clinical specimens in other units during and following this cluster.

During the period from April 2015 to March 2021, a total of eleven cases of IC caused by *Candida* species other than FRC were identified, comprising six cases caused by fluconazole-susceptible *C. parapsilosis*, four caused by *C. albicans*, and one caused by *C. tropicalis*. The incidence rates of the IC before the cluster, during the cluster, and after the cluster were not significantly different (Supplementary Table 3).

### Hand hygiene education

Before the implementation of the HH education, the overall HHC rate was comparable to baseline (94/128 [73%] vs 82/116 [71%] opportunities, *P* ≥ .05) (Table 1). However, the HHC rate before performing a sterile or aseptic procedure was significantly lower than the baseline (11/14 [79%] vs 3/12 [25%] opportunities, *P* < .05). After the education, the rate was increased, reaching levels comparable to that at the baseline (11/14 [79%] vs 8/12 [67%] opportunities, *P* ≥ .05).

**Table 1.** Hand hygiene compliance rates of the staff of the neonatal intensive care unit

	HHC rate (the number of observed HH actions/the total number of opportunities)							
	Overall	Physician	Nurse	WHO’s five moments for HH				
				Before touching a patient	Before performing a sterile or aseptic procedure	After potential exposure to body fluids	After touching a patient	After touching the patient’s immediate environment
May 18, 2018 (Baseline)	94/128 (73%)	10/14 (71%)	83/108 (77%)	28/34 (82%)	11/14 (79%)	3/7 (43%)	21/28 (75%)	20/25 (80%)
June 28, 2019 (Before HH education)	82/116 (71%)	12/25 (48%)	69/89 (78%)	32/42 (76%)	3/12 (25%)^a^	4/7 (57%)	27/33 (82%)	16/22 (73%)
November 27, 2019 (After HH education)	107/132 (81%)	10/19 (53%)	95/105 (90%)^a^	42/48 (88%)	8/12 (67%)	3/3 (100%)	36/45 (80%)	18/24 (75%)
May 19, 2020 (After the cluster)	104/131 (79%)	8/13 (62%)	96/118 (81%)	33/40 (83%)	14/23 (61%)	4/6 (67%)	31/37 (84%)	22/25 (88%)
November 19, 2020 (After the cluster)	103/147 (70%)	10/19 (53%)	92/127 (72%)	37/53 (70%)	19/28 (68%)	5/7 (71%)	18/28 (64%)	24/31 (77%)

Note. Abbreviations: WHO, World Health Organization; HHC, hand-hygiene compliance; HH, hand hygiene.

a Statistically significant difference from the baseline (*P* < .05).

The HHC rate in physicians was significantly worse than that in nurses both before and after the HH education (12/25 [48%] vs 69/89 [78%] and 10/19 [53%] vs 95/105 [90%] opportunities, respectively; both *P* < .05). After the HH education, the HHC rate in nurses significantly improved (83/108 [77%] vs 95/105 [90%] opportunities, *P* < .05), whereas that in physicians remained unchanged (10/14 [71%] vs 10/19 [53%] opportunities, *P* ≥ .05).

On the direct observation on November 19, 2020, after the cluster, the overall HHC rate was observed to be similar to that at the baseline level (94/128 [73%] vs 103/147 [71%] opportunities, *P* ≥ .05). However, there was a noticeable decline in the HHC rate among nurses, reverting to baseline levels (83/108 [77%] vs 92/127 [72%] opportunities, *P* ≥ .05).

### Environmental investigation

*C. parapsilosis* was detected in 5 of the 35 environmental cultures, which included samples from a medical device (diaper scale) in the NICU, three sinks (Nos. 1 to 3) in the NICU, and one sink (No.12) in the milk preparation room (Figure 2). It was not detected in sink No.4 used for infusion disposal. As shown in Figure 2, FRC strains were isolated from two sink surfaces/drains (sink No.1 and 3) near NICU beds No.1 through 4, which were occupied by 13 out of 14 patients (93%) at admission. Other *Candida* species including *C. albicans*, *C. guilliermondii*, *C. intermedia*, and *C. lusitaniae* were detected in 9 out of 12 cultures (75%) from sink drains and were not detected in 9 cultures from high-touch surfaces, 10 from medical devices or 4 from an incubator.

### Antifungal susceptibility testing

The MICs of all the FRC strains, comprising 2 isolated from the environment and 14 derived from patients, as determined by the fully automated analyzer, were further confirmed using the CLSI method. These strains with MIC values of ≤2 were found to be susceptible to fluconazole (Figure 2). Although no standard quantitative definition exists, the trailing effect is often defined as the presence of residual growth in wells containing antifungal agents at concentrations above the MIC.^12,13^ These isolates showed residual growth in wells containing 32 μg/mL of fluconazole using broth microdilution procedures, indicating the trailing effect.^12^ All *C. parapsilosis* isolates with MIC values of ≤2, including the fluconazole-trailing strains, were susceptible to amphotericin B and micafungin, as determined by the automated analyzer.

### Phylogenetic analysis

**Figure 3.**
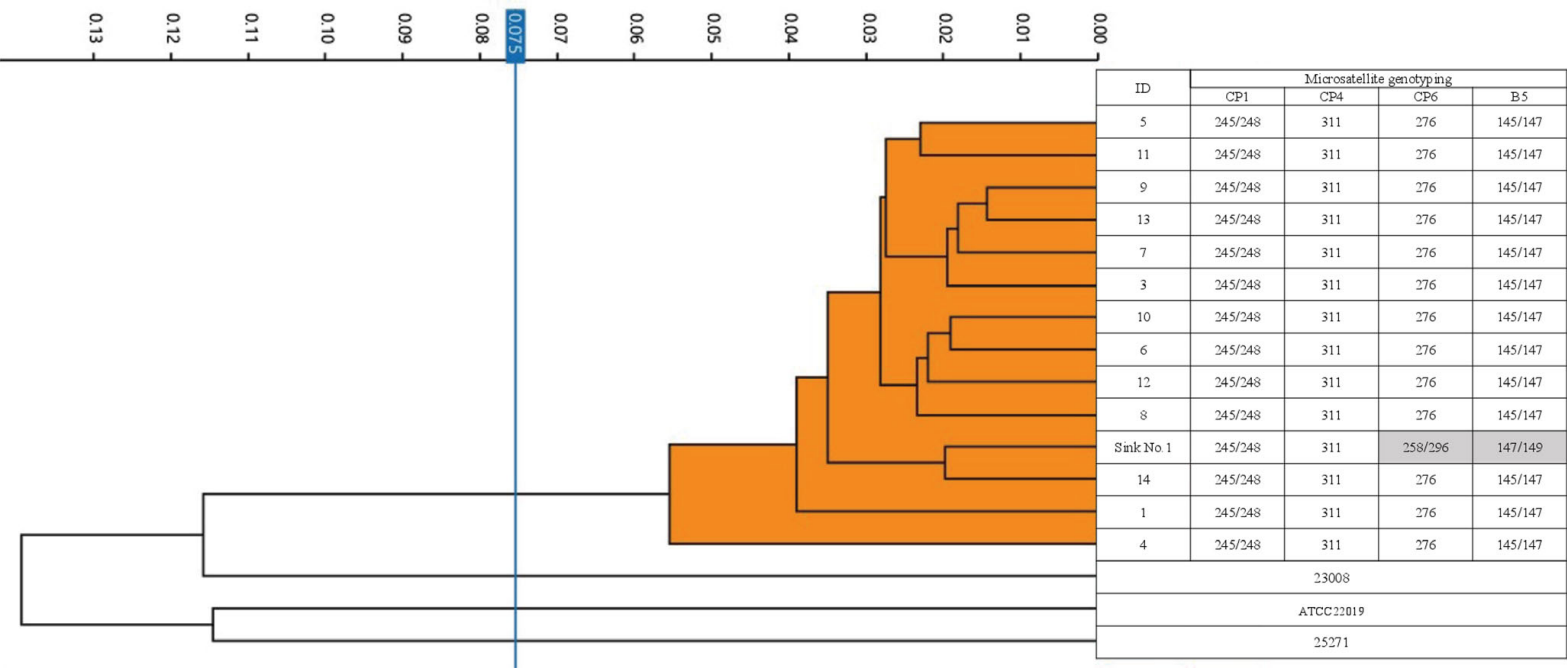
Fourier-transform infrared spectroscopy-based dendrogram of the one standard strain of *C. parapsilosis* (ATCC22019) and two epidemiologically unrelated clinical isolates from the university hospital (23008 and 25271), microsatellite alleles and fluconazole susceptibility of the *Candida parapsilosis* trailing strain. The blue line represents the automatic cutoff value (0.075). Clusters composed of the trailing strains are shaded orange. Gray boxes indicate the allele numbers that differ from the others.

Phylogenetic analysis utilizing FT-IR spectroscopy was performed on a total of 14 fluconazole-trailing strains, comprising 13 isolates derived from patients and one isolated from sink No.1 (Figure 3). Strains derived from patient No.2 and sink No.3 were excluded due to lack of preservation. The phylogenetic analysis showed that all the included isolates were clustered together and separated from epidemiologically unrelated strains, including ATCC 22019 (Figure 3). A cutoff value of 0.075 was determined for cluster attribution. Microsatellite genotyping also indicated that all pseudo-resistant isolates from patients had the same genotype; however, an isolate from sink No.1 was assigned to a genotype different from the other patient-derived isolates (Figure 3).

### Environmental infection control

To enhance environmental infection control, the following comprehensive interventions were implemented. Infection control nurses checked the cleaning practices of high-touch environmental surfaces to ensure thorough compliance. In September 2019 and January 2020, sinks with *Candida* species were disinfected using a commercially available 0.5% accelerated hydrogen peroxide (AHP)-based disinfectant (Hyprox accele^TM^, Virox Technologies Inc., Oakville, ON, Canada). Hydrogen peroxide, including AHP, has been demonstrated to be safe for both humans and the environment.^14^ Its germicidal efficacy against fungi including *Candida* species has been widely documented.^15–17^ The Centers for Disease Control and Prevention guidelines include AHP as an effective agent for disinfecting environmental surfaces.^17^ The sink bowls were covered with Virox-soaked gauze for 5 min,^17^ then wiped with clean gauze. The drains were flushed with Virox and drainage pipes (tailpieces, traps, and drain extensions) were replaced. After disinfection, the resampled cultures from the sink drains showed no fungal species. Additionally, three 10-min cycles of Ultraviolet-C (UV-C) irradiation using MoonBeam^TM^3 (Diversey, Fort Mill, SC, USA) were applied in the milk preparation room in January 2020.

## Discussion

This study describes the successful containment of a cluster of IC caused by fluconazole-trailing *C. parapsilosis* in the NICU using multiple infection-control interventions. The study highlights the role of sink drains as important reservoirs for *C. parapsilosis*, underlining the importance of maintaining HHC in healthcare providers, environmental hygiene, and regular monitoring to prevent nosocomial transmission in the NICU.

According to a previous systematic review, lower incidence rates of hospital-acquired infection can be achieved with HHC rates of over 60%.^18^ During the study period, the overall HHC rate consistently exceeded 70%, although the possibility of overestimation due to the Hawthorne effect cannot be disregarded.^19^ Notably, the rate before performing a sterile or aseptic procedure was significantly lower at 25% before the HH education. The observed low HHC rate before performing a sterile or aseptic procedure might have been a contributing factor to the cluster, including three cases of catheter-related BSI. Furthermore, the HHC rates in physicians were markedly lower than those in nurses as previously reported,^20,21^ averaging around 50%, and showed no significant improvement after HH education. Conversely, the HHC rate in nurses improved significantly to 90% following the education but regressed to 72% after 1 year, returning to the baseline level. Despite the lower number of observed HH opportunities for physicians compared to nurses, and the variability in the number of opportunities among the WHO’s five moments for HH, this study demonstrated that the HH education should be prioritized for physicians and the appropriate moments for HH, and should be implemented regularly to maintain high HHC rate.

Environmental investigations revealed that only sinks near the incubators used by patients infected or colonized with a fluconazole-trailing *C. parapsilosis* strain at admission were contaminated with that strain, suggesting that the sinks were the source of the cluster. Sinks are a major reservoir of pathogenic microorganisms.^22,23^ Previous studies have indicated a correlation between nosocomial infection of *C. parapsilosis* and contaminated water environments.^2,24^ *Candida* species that colonize sink drains are frequently dispersed through the splattering of water, and this dispersion may lead to the contamination of medical devices or the hands of healthcare providers.^25^ *C. parapsilosis* is a common cause of outbreaks in NICUs because it forms biofilms that adhere to environmental surfaces and medical devices and is spread via the hands of healthcare providers.^2,26,27^ Therefore, in addition to monitoring and preventing contamination of sinks with *C. parapsilosis,* maintaining HHC in healthcare providers is crucial in preventing the acquisition of *C. parapsilosis* and the occurrence of IC in the NICU.

While it can be challenging to entirely eradicate pathogens from contaminated sinks, previous studies have demonstrated that implementing risk mitigation measures, such as sink cleaning and disinfection, is effective in interrupting clusters.^28^ In this study, sink surfaces contaminated with *Candida* species were successfully decontaminated using AHP. Furthermore, UV-C irradiation was applied in the milk preparation room, predicated on the hypothesis of potential environmental contamination. The milk preparation room, utilized for both formula and donor milk preparation by nursing staff and expression of milk by mothers, presents a risk for pathogen transmission to infants through milk, milk preparation equipment, and environmental surfaces when milk handling procedures and HH practices are inadequate.^29^ UV-C devices are frequently used as an adjunct to standard cleaning in healthcare settings and are effective against *Candida* species on environmental surfaces.^30,31^ Meanwhile, the most effective cluster mitigation strategy involves the replacement of sink components, despite the costs and labor-intensive nature.^28^ In this study, the drainage pipes, which are particularly challenging to decontaminate using disinfectants, were replaced.

Owing to the trailing effect, the automated analyzer can occasionally misread the MIC and report pseudo-resistance.^13,32^ Thus, confirmation might be required when high MICs are observed, particularly in regions where resistant strains are uncommon. To date, knowledge regarding the molecular mechanisms responsible for the trailing effect is limited, and the clinical implications of the trailing effect remain unclear.^12,33^ In this study, four out of five infants infected with the fluconazole-trailing *C. parapsilosis* strain were successfully treated with amphotericin B or micafungin. One case of peritonitis resulted in fatality despite combined treatment with amphotericin B and fluconazole, although no definitive evidence indicated that fungal infection directly contributed to the cause of death. In premature infants, who are particularly susceptible to IC, the potential influence of the trailing effect should be considered when selecting antifungal agents, as it may affect their efficacy.^12^

Strain typing of *Candida* species is useful for identifying nosocomial transmission by elucidating the clonality among strains, and for determining the infection route by linking clinical isolates with environmental reservoirs.^2^ Although several discriminating molecular typing methods have been developed for *Candida* spp., including random amplified polymorphic deoxyribonucleic acid typing and amplified fragment length polymorphism fingerprinting, these methods require highly trained staff and are time-consuming, expensive, and unsuitable for real-time surveillance.^24,34^ In this study, phylogenetic analysis based on FT-IR spectroscopy helped detect the clonal spread of a fluconazole pseudo-resistant strain among patients. This finding was substantiated through microsatellite genotyping. While not considered the gold standard, this method represents the most widely used high-resolution approach for typing *C. parapsilosis*.^35^ FT-IR spectroscopy has previously proven to be useful for typing bacterial and fungal species (including *C. albicans* and *C. auris*) to investigate hospital outbreaks.^34,36^ To our knowledge, however, this is the first study to examine its application to *C. parapsilosis*. In this study, the results obtained through FT-IR spectroscopy and microsatellite genotyping were consistent for the patient-derived isolates, whereas discordance was observed for the environment-derived isolates. FT-IR spectra can fluctuate depending on the conditions during cultivation and sample preparation, and a standard method for the phylogenetic analysis of *C. parapsilosis* has not yet been established.^34,36^ Although further optimization and evaluation are needed to enhance its discriminatory power, FT-IR spectroscopy is rapid, easy to use, and inexpensive compared to other typing methods.^34^ FT-IR spectroscopy is a promising tool for the early detection of nosocomial transmission of *C. parapsilosis*.

Our study had some limitations. First, owing to the simultaneous implementation of multiple measures in the study, it was challenging to ascertain which measure exhibited the highest efficacy. Second, the cause of the trailing presented by *C. parapsilosis* in this study is unknown because molecular investigations related to antifungal susceptibility have not been conducted. Third, the transmission of *C. parapsilosis* via the hands of healthcare providers remains unconfirmed because we did not examine the contamination of healthcare providers. Nevertheless, a cluster of invasive candidiasis caused by fluconazole-trailing *C. parapsilosis,* presenting with fluconazole trailing, in the NICU was successfully treated by implementing targeted infection-control interventions for contaminated sinks, identified using an environmental investigation, and the milk preparation room, and by improving HHC among NICU staff. Ensuring environmental hygiene and constant staff education to maintain a high HHC level is necessary to limit the spread of *C. parapsilosis* strains in the NICU.

## Supporting information

Baba et al. supplementary material Baba et al. supplementary material

## Supplementary material

For supplementary material accompanying this paper visit http://doi.org/10.1017/ash.2024.77. click here to view supplementary material

## References

[1] Weimer KED , Smith PB , Puia-Dumitrescu M , Aleem S. Invasive fungal infections in neonates: a review. Pediatr Res 2022;91:404–412. 34880444 10.1038/s41390-021-01842-7

[2] Miyake A , Gotoh K , Iwahashi J , et al. Characteristics of biofilms formed by C. parapsilosis causing an outbreak in a neonatal intensive care unit. J Fungi (Basel) 2022;8:700. 35887456 10.3390/jof8070700 PMC9322970

[3] Piqueras A , Ganapathi L , Carpenter JF , et al. Trends in pediatric candidemia: epidemiology, anti-fungal susceptibility, and patient characteristics in a children’s hospital. J Fungi (Basel) 2021;7:78 33499285 10.3390/jof7020078 PMC7911199

[4] Trevijano-Contador N , Torres-Cano A , Carballo-González C , et al. Global emergence of resistance to fluconazole and voriconazole in *Candida parapsilosis* in tertiary hospitals in Spain during the COVID-19 pandemic. Open Forum Infect Dis 2022;9:ofac605. 36467290 10.1093/ofid/ofac605 PMC9709632

[5] Stone SP , Cooper BS , Kibbler CC , et al. The ORION statement: guidelines for transparent reporting of outbreak reports and intervention studies of nosocomial infection. Lancet Infect Dis 2007;7:282–288. 17376385 10.1016/S1473-3099(07)70082-8

[6] Pinhat EC , Borba MGS , Ferreira ML , et al. Fungal colonization in newborn babies of very low birth weight: a cohort study. J Pediatr (Rio J) 2012;88:211–216. 22622625 10.2223/JPED.2192

[7] Siegel D , Rhinehart E , Jackson M , Chiarello L , The Healthcare Infection Control Practices Advisory Committee. 2007 Guideline for isolation precautions: preventing transmission of infectious agents in healthcare settings. Am J Infect Control 2007;35:S65–164. 18068815 10.1016/j.ajic.2007.10.007 PMC7119119

[8] World Health Organization. WHO Guidelines on Hand Hygiene in Health Care. Geneva: World Health Organization Press; 2009.

[9] Clinical and Laboratory Standards Institute. Reference Method for Broth Dilution Antifungal Susceptibility Testing of Yeasts, 4th edition. Wayne, PA: CLSI; 2017.

[10] Bruker Daltonics GmbH & Co. KG. Instructions for Use IR Biotyper Kit. https://www.bruker.com/en/resources/certificates-data-sheets/ifu.html?q_1=IR-Biotyper. Published 2021. Accessed January 17, 2024.

[11] Sabino R , Sampaio P , Rosado L , Stevens DA , Clemons KV , Pais C. New polymorphic microsatellite markers able to distinguish among *Candida parapsilosis sensu stricto* isolates. J Clin Microbiol 2010;48:1677–1682. 20220157 10.1128/JCM.02151-09 PMC2863883

[12] Rueda C , Puig-Asensio M , Guinea J , et al. Evaluation of the possible influence of trailing and paradoxical effects on the clinical outcome of patients with candidemia. Clin Microbiol Infect 2017;23:49.e1–49.e8. 10.1016/j.cmi.2016.09.016 27677697

[13] Zhang L , Wang H , Xiao M , et al. The widely used ATB FUNGUS 3 automated readings in China and its misleading high MICs of *Candida* spp. to azoles: challenges for developing countries’ clinical microbiology labs. PLoS One 2014;9:e114004. 25460351 10.1371/journal.pone.0114004 PMC4252076

[14] Omidbakhsh N , Sattar SA. Broad-spectrum microbicidal activity, toxicologic assessment, and materials compatibility of a new generation of accelerated hydrogen peroxide-based environmental surface disinfectant. Am J Infect Control 2006;34:251–257. 16765201 10.1016/j.ajic.2005.06.002 PMC7132737

[15] Rutala WA , Kanamori H , Gergen MF , Sickbert-Bennett EE , Weber DJ. Susceptibility of *Candida auris* and *Candida albicans* to 21 germicides used in healthcare facilities. Infect Control Hosp Epidemiol 2019;40:380–382. 30767810 10.1017/ice.2019.1

[16] Cadnum JL , Shaikh AA , Piedrahita CT , et al. Effectiveness of disinfectants against *Candida auris* and other *Candida* species. Infect Control Hosp Epidemiol. 2017;38:1240–1243. 28793937 10.1017/ice.2017.162

[17] Rutala W , Weber D , The Healthcare Infection Control Practices Advisory Committee (HICPAC). Guideline for Disinfection and Sterilization in Healthcare Facilities, 2008. https://www.cdc.gov/infectioncontrol/guidelines/disinfection/. Published 2019. Accessed November 9, 2023.

[18] Mouajou V , Adams K , DeLisle G , Quach C. Hand hygiene compliance in the prevention of hospital-acquired infections: a systematic review. J Hosp Infect 2022;119:33–48. 34582962 10.1016/j.jhin.2021.09.016

[19] McCambridge J , Witton J , Elbourne DR. Systematic review of the Hawthorne effect: new concepts are needed to study research participation effects. J Clin Epidemiol 2014;67:267–277. 24275499 10.1016/j.jclinepi.2013.08.015 PMC3969247

[20] Ojanperä H , Ohtonen P , Kanste O , Syrjälä H. Impact of direct hand hygiene observations and feedback on hand hygiene compliance among nurses and doctors in medical and surgical wards: an eight-year observational study. J Hosp Infect. 2022;127:83–90. 35724953 10.1016/j.jhin.2022.06.007

[21] Bredin D , O’Doherty D , Hannigan A , Kingston L. Hand hygiene compliance by direct observation in physicians and nurses: a systematic review and meta-analysis. J Hosp Infect 2022;130:20–33. 36089071 10.1016/j.jhin.2022.08.013

[22] Kanamori H , Weber DJ , Rutala WA. Healthcare outbreaks associated with a water reservoir and infection prevention strategies. Clin Infect Dis 2016;62:1423–1435. 26936670 10.1093/cid/ciw122

[23] Baba H , Kanamori H , Katsumi M , et al. A case of meningitis due to extensively drug-resistant *Pseudomonas aeruginosa* imported through medical evacuation: genomic and environmental investigation. J Travel Med 2021;28:taab047. 33763694 10.1093/jtm/taab047

[24] Qi L , Fan W , Xia X , et al. Nosocomial outbreak of *Candida parapsilosis sensu stricto* fungaemia in a neonatal intensive care unit in China. J Hosp Infect 2018;100:e246–e252. 29928941 10.1016/j.jhin.2018.06.009

[25] Jencson AL , Cadnum JL , Piedrahita C , Donskey CJ. Hospital sinks are a potential nosocomial source of Candida infections. Clin Infect Dis 2017;65:1954–1955. 29020154 10.1093/cid/cix629

[26] Pammi M , Holland L , Butler G , Gacser A , Bliss JM. *Candida parapsilosis* is a significant neonatal pathogen. Pediatr Infect Dis J 2013;32:e206–e216. 23340551 10.1097/INF.0b013e3182863a1c PMC3681839

[27] van Asbeck EC , Huang YC , Markham AN , Clemons KV , Stevens DA. *Candida parapsilosis* fungemia in neonates: genotyping results suggest healthcare workers hands as source, and review of published studies. Mycopathologia 2007;164:287–293. 17874281 10.1007/s11046-007-9054-3

[28] Parkes LO , Hota SS. Sink-related outbreaks and mitigation strategies in healthcare facilities. Curr Infect Dis Rep 2018;20:42. 30128678 10.1007/s11908-018-0648-3

[29] Antimicrobial Resistance and Healthcare Associated Infection (ARHAI) Scotland Infection Control team. Literature Review and Practice Recommendations: Management of Incidents and Outbreaks in Neonatal Units (NNUs). https://www.nipcm.hps.scot.nhs.uk/media/1886/2022-06-16-mngment-of-incidents-and-outbreaks-in-nnus-v20.pdf. Published 2022. Accessed November 13, 2023.

[30] Rutala WA , Kanamori H , Gergen MF , Sickbert-Bennett EE , Weber DJ. Inactivation of *Candida auris* and *Candida albicans* by ultraviolet-C. Infect Control Hosp Epidemiol 2022;43:1495–1497. 34016204 10.1017/ice.2021.214

[31] Cadnum JL , Shaikh AA , Piedrahita CT , et al. Relative resistance of the emerging fungal pathogen *Candida auris* and other *Candida* species to killing by ultraviolet light. Infect Control Hosp Epidemiol 2018;39:94–96. 29157326 10.1017/ice.2017.239

[32] Ono T , Suematsu H , Sawamura H , Yamagishi Y , Mikamo H. Confirming the utility of RAISUS antifungal susceptibility testing by new-software. Rinsho Biseibutshu Jinsoku Shindan Kenkyukai Shi 2017;27:47–56. 28817940

[33] Marcos-Zambrano LJ , Escribano P , Sánchez-Carrillo C , Bouza E , Guinea J. Scope and frequency of fluconazole trailing assessed using EUCAST in invasive *Candida spp.* isolates. Med Mycol 2016;54:733–739. 27161788 10.1093/mmy/myw033

[34] Vatanshenassan M , Boekhout T , Mauder N , et al. Evaluation of microsatellite typing, ITS sequencing, AFLP fingerprinting, MALDI-TOF MS, and fourier-transform infrared spectroscopy analysis of *Candida auris* . J Fungi (Basel) 2020;6:146. 32854308 10.3390/jof6030146 PMC7576496

[35] Sabino R , Sampaio P , Rosado L , Videira Z , Grenouillet F , Pais C. Analysis of clinical and environmental *Candida parapsilosis* isolates by microsatellite genotyping--a tool for hospital infection surveillance. Clin Microbiol Infect 2015;21:954.e1–8. 10.1016/j.cmi.2015.06.001 26070962

[36] Sandt C , Sockalingum GD , Aubert D , et al. Use of Fourier-transform infrared spectroscopy for typing of *Candida albicans* strains isolated in intensive care units. J Clin Microbiol 2003;41:954–959. 12624015 10.1128/JCM.41.3.954-959.2003 PMC150280

