# Supplementary material for: A cluster of Candida parapsilosis displaying fluconazole-trailing in a neonatal intensive care unit successfully contained by multiple infection-control interventions
Source: Antimicrob Steward Healthc Epidemiol. 2024 May 16;4(1):e86. doi: 10.1017/ash.2024.77 (PMC11106732; doi:10.1017/ash.2024.77)

**Supplementary Method 1. Identification of fungal strains.** The clinical and environmental samples were inoculated onto Chromogram™ Candida media (Kanto Chemical Co., Tokyo, Japan) and incubated at 37°C for 48 h. Colonies were identified based on morphology, and a representative colony was selected and stored in 10% skimmed milk at -30℃.

**Supplementary Method 2. Microsatellite genotyping.** Genomic DNA of all the fluconazole-resistant *Candida parapsilosis* strains was extracted by the Single-tube LiOAc-SDS lysis method^1^. The four loci, CP1, CP4, CP6, and B5, were amplified using monoplex PCR. After PCR, amplicons were diluted 10-fold with distilled water, and 1µl aliquots were added to 9.5 µL formamide and 0.5 µL internal size standard (Internal Lane Standard 600; Promega). Samples were denatured at 95°C for 5 min and cooled rapidly on ice. Samples were analyzed by an ABI Prism 3130 Genetic analyzer (Applied Biosystems). The sizes of these amplicons were determined by using Peak Scanner (version 2) analysis software (https://www.thermofisher.com/order/catalog/product/4381867).

Supplementary References

1. Lõoke M, Kristjuhan K, Kristjuhan A. Extraction of genomic DNA from yeasts for PCR-based applications. *Biotechniques*. 2011;50(5):325-328.

**Supplementary Table 1. The clinical setting, population characteristics, and infection control interventions associated with the fluconazole-resistant *Candida parapsilosis* cluster.**

| Setting | A neonatal care unit (15 beds) and a growing care unit (17 beds) in a tertiary hospital with 900 deliveries and hospitalizations involving 30 very low birth weight and 40 extremely low birth weight infants |
| --- | --- |
| Dates | April 2019-March 2020 |
| Population characteristics | A total of 201 newborns admitted to the NICU (mean birth weight=2,134 g; gestational age=34 weeks) |
| FRC screening policy | Admission and weekly cultures from rectal, nasal, and skin samples |
| Interventions | Active surveillance (May 1, 2019, to March 31, 2020) |
|  | Hand hygiene education for NICU staff (July 2019) |
|  | Environmental investigation (September 18, 2019) |
|  | Disinfection interventions on sink surface/drains (September 30, 2019, and January 22, 2020) |

Abbreviations: FRC, fluconazole-resistant *Candida parapsilosis***.**

**Supplementary Table 2. A retrospective case-control study conducted on all patients admitted to the NICU from April to August 2019.** Patients infected or colonized with fluconazole-resistant *C. parapsilosis* were categolized as cases (n=11) and all other patients as controls (n=26).

| Patient characteristics | Cases n=11 | Controls n=26 | OR | 95% CI | *p*-value |
| --- | --- | --- | --- | --- | --- |
| Place of birth = inborn (%) | 11 (100%) | 25 (96%) | NA | NA | >0.05 |
| Caesarean section = yes (%) | 11 (100%) | 25 (96%) | NA | NA | >0.05 |
| Maternal Candida vaginitis = yes (%) | 0 (0%) | 0 (0%) | NA | NA | >0.05 |
| Prelabor rupture of membrane = yes (%) | 4 (36%) | 8 (31%) | 1.29 | 0.29-5.67 | >0.05 |
| Antenatal steroids = yes (%) | 5 (45%) | 11 (42%) | 1.14 | 0.27-4.7 | >0.05 |
| Antenatal antibiotics = yes (%) | 6 (55%) | 9 (35%) | 2.27 | 0.54-9.53 | >0.05 |
| Incubator = yes (%) | 11 (100%) | 26 (100%) | NA | NA | >0.05 |
| Mother’s milk = yes (%) | 11 (100%) | 26 (100%) | NA | NA | >0.05 |
| Donor milk = yes (%) | 9 (82%) | 10 (38%) | 7.20 | 1.28-40.37 | 0.029 |
| Formula = yes (%) | 6 (55%) | 25 (96%) | 0.05 | 0.00-0.49 | 0.005 |
| Total parenteral nutrition = yes (%) | 11 (100%) | 21 (81%) | NA | NA | >0.05 |
| Peripheral venous catheter = yes (%) | 10 (91%) | 18 (69%) | 4.44 | 0.48-40.84 | >0.05 |
| Central venous catheter = yes (%) | 11 (100%) | 23 (88%) | NA | NA | >0.05 |
| Umbilical catheter = yes (%) | 1 (9%) | 26 (100%) | NA | NA | >0.05 |
| Surgery = yes (%) | 3 (27%) | 10 (38%) | 0.60 | 0.13-2.81 | >0.05 |
| Respirator = yes (%) | 11 (100%) | 23 (88%) | NA | NA | >0.05 |
| NPPV = yes (%) | 4 (36%) | 22 (85%) | 0.10 | 0.02-0.53 | 0.006 |
| X-ray = yes (%) | 11 (100%) | 25 (96%) | NA | NA | >0.05 |
| Ultrasound = yes (%) | 11 (100%) | 26 (100%) | NA | NA | >0.05 |
| Antibiotics during admission = yes (%) | 11 (100%) | 21 (81%) | NA | NA | >0.05 |
| Antifungal prophylaxis = yes (%) | 0 (0%) | 0 (0%) | NA | NA | >0.05 |

Categorical variables were compared between cases and controls using Fisher’s exact test. Analysis was performed using JMP Pro 16 statistical analysis software (SAS Institute, 2021).

Abbreviations: OR, odds ratio; CI, confidence interval; SD, standard deviation; NA, not applicable; NPPV, non-invasive positive pressure ventilation.

**Supplementary Table 3. The incidence rates of invasive candidiasis caused by *Candida* species other than fluconazole-resistant *Candida parapsilosis*.**

|  | | Before the cluster | | | | During the cluster | After the cluster |
| --- | --- | --- | --- | --- | --- | --- | --- |
|  |  | April 2015 to March 2016 | April 2016 to March 2017 | April 2017 to March 2018 | April 2018 to March 2019 | April 2019 to March 2020 | April 2020 to March 2021 |
| Total | Inpatients (n) | 311 | 260 | 231 | 245 | 232 | 204 |
|  | IC cases (n) | 4 | 1 | 1 | 0 | 3 | 2 |
|  | Incidence rates (%) | 1.3 | 0.4 | 0.4 | 0 | 1.3 | 1.0 |
| BW  500-1,000g | Inpatients (n) | 34 | 24 | 37 | 33 | 28 | 27 |
|  | IC cases (n) | 2 | 0 | 1 | 0 | 1 | 0 |
|  | Incidence rates (%) | 5.9 | 0 | 2.7 | 0 | 3.6 | 0 |
| BW  <500g | Inpatients (n) | 6 | 11 | 9 | 9 | 7 | 8 |
|  | IC cases (n) | 1 | 1 | 0 | 0 | 2 | 2 |
|  | Incidence rates (%) | 16.7 | 9.1 | 0 | 0 | 28.6 | 25.0 |

Categorical variables were compared using Fisher’s exact test. Analysis was performed using JMP Pro 16 statistical analysis software (SAS Institute, 2021). The incidence rates of the invasive candidiasis before the cluster, during the cluster, and after the cluster were not significantly different: for total inpatients, the rates were 0.6% (0/1047), 1.3% (3/232), and 1.0% (2/204); for inpatients with a birthweight 500-1,000g, the rates were 2.3% (3/128), 3.6% (1/28), and 0% (0/27); and for inpatients with a birthweight below 500g, the rates were 5.7% (2/35), 28.6% (2/7), and 25.0% (2/8), respectively, all with *p*≥0.05.

Abbreviations: BW, birthweight; IC, invasive candidiasis.

**Supplementary Figure. The standard sinks in the NICU.**


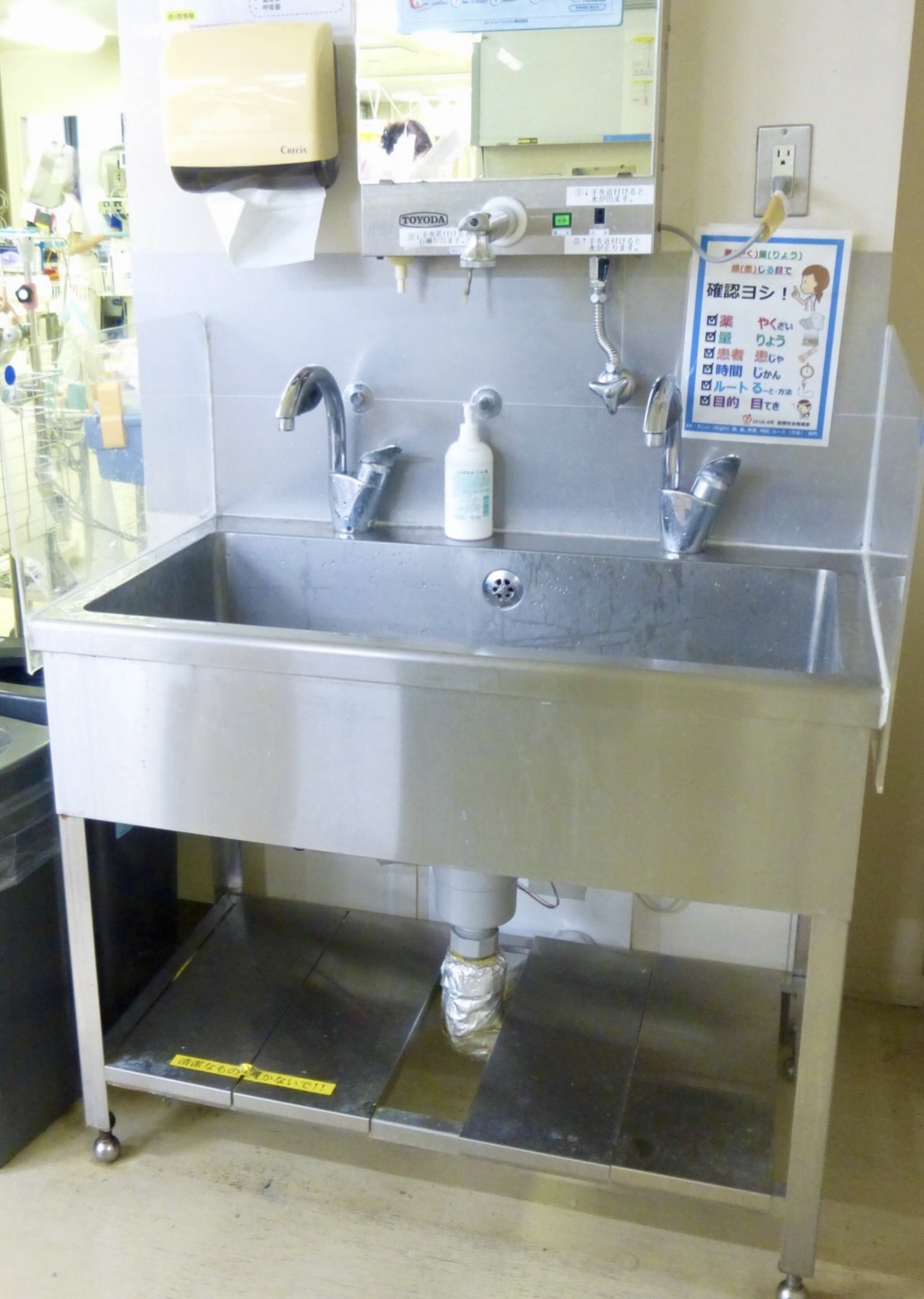

Supplement: Baba et al. supplementary material [file S2732494X24000779sup001.docx]
